# Impact of Age and Diastolic Function on Novel, 4D flow CMR Biomarkers of Left Ventricular Blood Flow Kinetic Energy

**DOI:** 10.1038/s41598-018-32707-5

**Published:** 2018-09-26

**Authors:** Saul Crandon, Jos J. M. Westenberg, Peter P. Swoboda, Graham J. Fent, James R. J. Foley, Pei G. Chew, Louise A. E. Brown, Christopher Saunderson, Abdallah Al-Mohammad, John P. Greenwood, Rob J. van der Geest, Erica Dall’Armellina, Sven Plein, Pankaj Garg

**Affiliations:** 1 Leeds Institute of Cardiovascular and Metabolic Medicine, University of Leeds, Leeds, UK; 2 Department of Radiology, Leiden University Medical Center, Leiden, The Netherlands; 3 Sheffield Teaching Hospitals NHS Foundation Trust, Sheffield, UK; 4 Department of Infection, Immunity & Cardiovascular Disease, University of Sheffield, Sheffield, United Kingdom

## Abstract

Two-dimensional (2D) methods of assessing mitral inflow velocities are pre-load dependent, limiting their reliability for evaluating diastolic function. Left ventricular (LV) blood flow kinetic energy (KE) derived from four-dimensional flow cardiovascular magnetic resonance imaging (4D flow CMR) may offer improvements. It remains unclear whether 4D LV blood flow KE parameters are associated with physiological factors, such as age when compared to 2D mitral inflow velocities. Fifty-three healthy volunteers underwent standard CMR, plus 4D flow acquisition. LV blood flow KE parameters demonstrated good reproducibility with mean coefficient of variation of 6 ± 2% and an accuracy of 99% with a precision of 97%. The LV blood flow KEi_EDV_ E/A ratio demonstrated good association to the 2D mitral inflow E/A ratio (r = 0.77, P < 0.01), with both decreasing progressively with advancing age (P < 0.01). Furthermore, peak E-wave KEi_EDV_ and A-wave KEi_EDV_ displayed a stronger association to age than the corresponding 2D metrics, peak E-wave and A-wave velocity (r = −0.51 vs −0.17 and r = 0.65 vs 0.46). Peak E-wave KEi_EDV_ decreases whilst peak A-wave KEi_EDV_ increases with advancing age. This study presents values for various LV blood flow KE parameters in health, as well as demonstrating that they show stronger and independent correlations to age than standard diastolic metrics.

## Introduction

Assessment of left ventricular (LV) diastolic function is an integral part of the routine evaluation of patients presenting with symptoms of shortness of breath or heart failure^1^. This is routinely done by Doppler echocardiography using mitral inflow velocities and tissue Doppler imaging (TDI). Even though cardiovascular magnetic resonance (CMR) imaging is the current gold standard for volumetric and tissue characterisation of the LV, it is infrequently used to assess LV diastolic function^2^. This is in part due to the impact of through-plane motion of routine two-dimensional (2D) phase contrast acquisition resulting in under-estimation of peak mitral inflow velocities and non-standardized techniques to estimate myocardial velocity^3^.

Four-dimensional (4D) flow CMR allows retrospective tracking of the mitral annulus and extrapolation of the dynamic phase contrast plane through the mitral valve, which can then be segmented to compute accurate mitral inflow velocities^4,5^. From the same 4D flow CMR dataset, a comprehensive assessment of the LV blood flow kinetic energy (KE) can also be made, by using a semi-automated method based on short-axis cine contours^6–12^. This three-dimensional flow assessment offers novel ways to assess the intra-ventricular flow in valvular disease as demonstrated in a study by Al-Wakeel *et al*. in which they show significant changes in LV blood flow KE in mitral regurgitation patients. Importantly, this study demonstrates a significant decrease of mean KE, systolic and early-diastolic KE peaks after mitral valve surgery^13^.

Assessment of LV blood flow KE by 4D flow CMR may offer similar efficacy to existing methods but may be less susceptible to inter-user variations and through-plane errors. It remains unclear if LV blood flow KE is associated with standard 2D parameters of diastolic function or patient characteristics like age, which are closely associated with myocardial stiffness^14,15^.

We hypothesize that KE markers will be closely associated to existing LV diastolic parameters. The aim of this study is to quantify different elements of LV blood flow KE using 4D flow CMR and investigate their association to CMR-derived 2D mitral inflow and myocardial tissue velocities. In addition, we aim to provide normal LV blood flow KE values for different age groups as well as comparing KE to standard diastolic parameters for their association with age.

## Results

### Demographic characteristics

All 53 healthy volunteers recruited completed the full study protocol. The recruited participants consisted of 32 males (60.3%) and 21 females (39.7%) with a mean age of 45 ± 17 years. A summary of the demographic characteristics of the study participants is provided in Table 1.

**Table 1:** Participant demographics, haemodynamic and kinetic energy (KE) variables for the overall study population, males and females.

Characteristic	Healthy volunteers (n = 53)	Males (n = 32)	Females (n = 21)	P value
Age (years)	45 ± 17	42 ± 17	50 ± 17	0.08
Body surface area (m^2^)	1.8 ± 0.2	1.9 ± 0.1	1.7 ± 0.2	<0.01
LVEDMi (g/m^2^)	52.2 ± 10.1	55.0 ± 10.1	48.0 ± 8.8	0.01
LVEDVi (ml/m^2^)	85.9 ± 18.1	85.9 ± 19.3	85.9 ± 16.7	0.99
LVESVi (ml/m^2^)	33.2 ± 10.2	33.2 ± 11.6	33.1 ± 7.9	0.96
SVi (ml/m^2^)	52.7 ± 9.8	52.6 ± 9.9	52.8 ± 9.8	0.96
EF (%)	61.8 ± 5.2	62.0 ± 6.0	61.6 ± 3.6	0.79
LV peak E-wave velocity*	76.7 ± 26.5	76.0 ± 23.1	77.8 ± 20.6	0.78
LV peak A-wave velocity*	51.0 ± 22.5	51.1 ± 19.4	51.0 ± 14.9	0.99
E/A ratio	1.6 ± 0.6	1.6 ± 0.6	1.7 ± 0.7	0.68
LV global KEi_EDV_^†^	8.7 ± 2.9	8.6 ± 3.7	8.7 ± 2.1	0.76
LV systolic KEi_EDV_^†^	9.8 ± 3.1	9.9 ± 2.6	8.8 ± 3.5	0.60
LV diastolic KEi_EDV_^†^	7.9 ± 3.8	7.7 ± 4.8	8.3 ± 3.0	0.48
LV peak E-wave KEi_EDV_^†^	23.2 ± 11.6	21.4 ± 12.1	25.4 ± 7.5	0.57
LV peak A-wave KEi_EDV_^†^	10.3 ± 8.4	9.7 ± 9.7	11.5 ± 6.5	0.60
KEi_EDV_ E/A ratio	2.6 ± 1.8	2.5 ± 2.3	2.5 ± 1.8	0.77

Demographic data is presented as mean ± standard deviation, whereas kinetic energy data is presented as median ± interquartile range.

*cm/s; ^**†**^μJ/ml, KE = kinetic energy of blood, LV = left ventricle, LVEDMi = left ventricular end-diastolic mass indexed, LVEDVi = left ventricular end-diastolic volume indexed, LVESVi = left ventricular end-systolic volume indexed, SVi = stroke volume indexed, EF = ejection fraction, KEi_EDV_ = kinetic energy indexed to end-diastolic volume.

### Baseline CMR data

Indexed left ventricular end-diastolic mass was significantly higher in males than females (mean ± SD, 55.0 ± 10.1 vs 48.0 ± 8.8 g/m^2^, P = 0.01). The sexes were matched on all other indexed CMR parameters. LV peak-E, peak-A velocities and E/A ratio were not significantly different in males versus females (Table 1).

### Normal values for 4D-flow derived diastolic parameters

#### Overall study population

Global LV KEi_EDV_ was 8.7 ± 2.9 μJ/ml (median ± IQR), with a systolic KEi_EDV_ of 9.8 ± 3.1 μJ/ml. Median diastolic KEi_EDV_ was 7.9 ± 3.8 μJ/ml, accompanied by a peak E-wave and peak A-wave KEi_EDV_ of 23.2 ± 11.6 and 10.3 ± 8.4 μJ/ml respectively, resulting in an average KEi_EDV_ E/A ratio of 2.6 ± 1.8.

#### Sex differences

No significant differences were present in the global LV KEi_EDV_ between males and females (8.6 ± 3.7 vs 8.7 ± 2.1 μJ/ml, P = 0.76). The same was also true for systolic and diastolic KEi_EDV_ (P = 0.60 and 0.48 respectively). Females exhibited a higher peak E-wave and A-wave KEi_EDV_, but a similar KEi_EDV_ E/A ratio (2.5 ± 1.8 vs. 2.5 ± 2.3, P = 0.77).

#### 2D mitral inflow metrics differences with age

**Figure 1:**
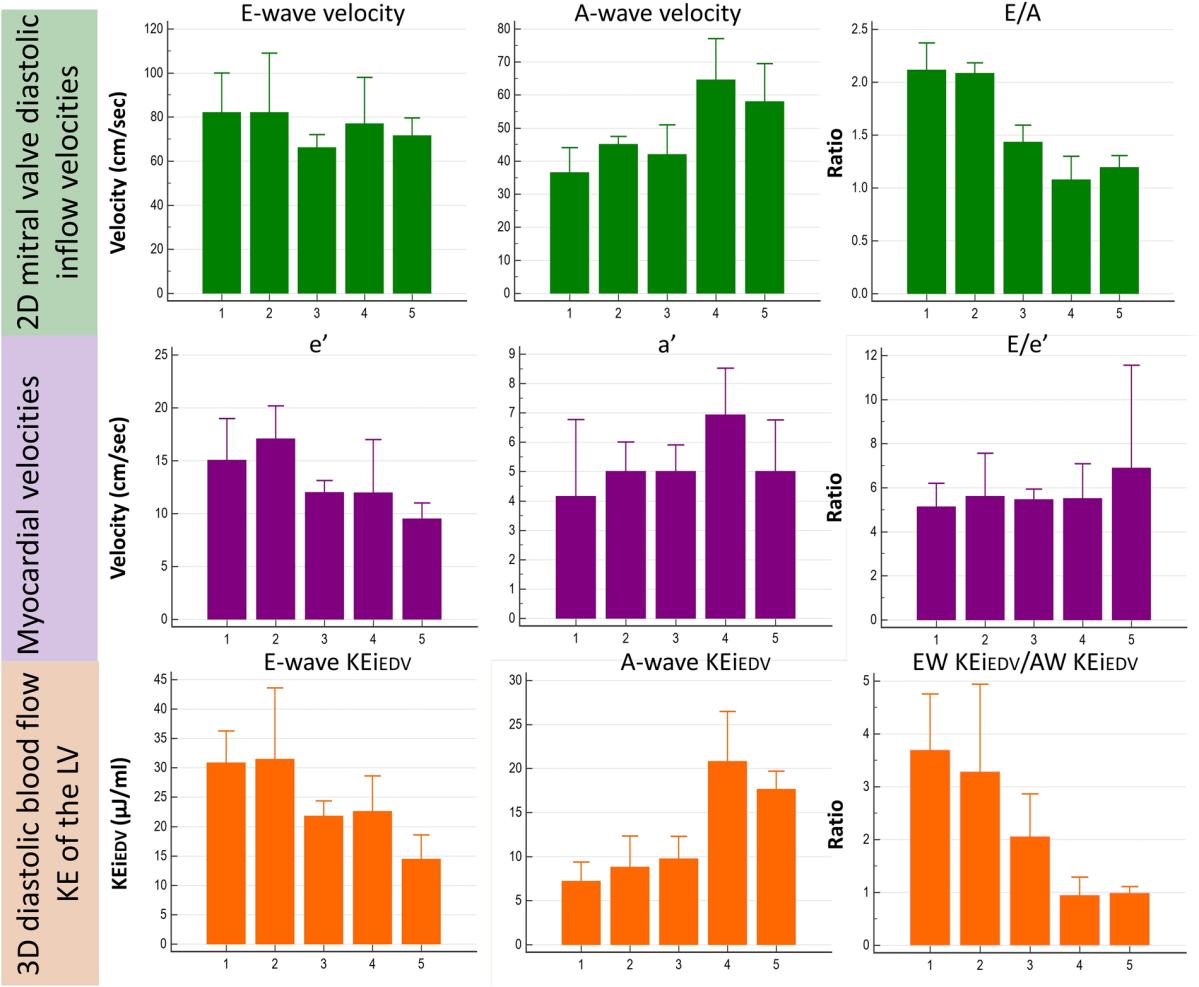
Bar chart displaying the reference values for 4D diastolic LV blood flow KE parameters along with myocardial velocities and 2D mitral valve diastolic inflow velocities. Advancing age is denoted on the x-axis, with the study population divided into groups (1–5). Group 1 = 23 ± 2 years old (n = 12), group 2 = 32 ± 3 (n = 9), group 3 = 47 ± 4 (n = 11), group 4 = 54 ± 2 (n = 10), group 5 = 69 ± 6 (n = 11). For the 2D mitral inflow velocities and myocardial velocities, the velocity (in cm/s) is given on the y-axis with errors bars denoting standard deviation (SD), whereas for 4D diastolic blood flow KE parameters, energy in μJ/ml is given, with error bars denoting interquartile range (IQR).

Peak E-wave and A-wave velocity were not significantly different amongst any of the 5 age groups (P > 0.05) (Fig. 1). Despite this, E/A ratio showed a significant decline with progressive age (P < 0.01), with further significance amongst the individual intergroup comparisons. Participants in groups 1 and 2 (aged ≤39 years) had a significantly higher E/A ratio than those in the older subgroups (P < 0.05).

#### 4D diastolic LV KE parameters differences with age

The differences in LV global and systolic KEi_EDV_ with age were non-significant. Increasing age resulted in a decline in peak E-wave KEi_EDV_ (P < 0.01), whilst peak A-wave KEi_EDV_ rose (P < 0.01). This resulted in a decrease in the KEi_EDV_ E/A ratio at higher ages (P < 0.01), displaying further inter-group significance. Participants aged over 50 years had a significantly lower KEi_EDV_ E/A ratio than all other younger age groups (P < 0.05). Table 2 provides a summary of the velocity and KE results for the various age groups.

**Table 2:** Post-hoc analysis of participant haemodynamic and kinetic energy (KE) variables (both indexed and non-indexed for end-diastolic volume) divided according to age groups.

Age groups (mean ± SD) years old	23 ± 2 (n = 12)^a^	32 ± 3 (n = 9)^b^	47 ± 4 (n = 11)^c^	54 ± 2 (n = 10)^d^	69 ± 6 (n = 11)^e^	P-value
LV peak E-wave velocity*	82.0 ± 35^c^	82 ± 35^c,d^	66 ± 15^a,b^	77 ± 35	71.5 ± 21^b^	0.04
LV peak A-wave velocity*	36.5 ± 13^d,e^	45 ± 6^d^	42 ± 14^d^	64.5 ± 21^a,b,c^	58 ± 30^a^	<0.01
E/A ratio	2.1 ± 0.9^c,d,e^	2 ± 0.5^c,d,e^	1.4 ± 0.3 ^a,b,d,e^	1.1 ± 0.3^a,b,c^	1.2 ± 0.4^a,b,c^	<0.01
LV global KEi_EDV_^†^	10 ± 2.4	9.48 ± 5	8 ± 3.4	9 ± 6.35	8 ± 1.3	0.06
LV systolic KEi_EDV_^†^	10 ± 3	11 ± 4	9 ± 3	10 ± 7	10 ± 2	0.84
LV diastolic KEi_EDV_^†^	9.7 ± 2.6^c,e^	10.6 ± 4^c,e^	8.17 ± 4^a,b,d^	9 ± 4.6^c,e^	7 ± 2.5^a,b,d^	0.01
LV peak E-wave KEi_EDV_^†^	30.8 ± 12^c,d,e^	31 ± 21^c,d,e^	22 ± 6^a,b^	23 ± 14^a,b,e^	14.4 ± 7^a,b,d^	<0.01
LV peak A-wave KEi_EDV_^†^	7.2 ± 3.5^d,e^	9 ± 6^d,e^	10 ± 6^d,e^	21 ± 13^a,b,c^	17.6 ± 6^a,b,c^	<0.01
KEi_EDV_ E/A ratio	3.7 ± 1.5^c,d,e^	3.3 ± 2.6^c,d,e^	2 ± 1^a,b,d,e^	0.94 ± 0.6^a,b,c^	0.98 ± 0.3^a,b,c^	<0.01
LV global KE^#^	1.6 ± 0.5	1.6 ± 0.8	1.5 ± 0.9	1.4 ± 0.4	1.2 ± 0.5	0.49
LV systolic KE^#^	1.7 ± 0.5	1.8 ± 1.4	1.7 ± 1	1.3 ± 0.3	1.3 ± 0.3	0.06
LV diastolic KE^#^	1.5 ± 0.8	1.2 ± 0.5	1.4 ± 1	1.4 ± 0.4	1 ± 0.7	0.77
LV peak E-wave KE^#^	3.6 ± 1.9	3.2 ± 2.1	3.5 ± 2.7	4.4 ± 1.8	2.6 ± 3.6	0.38
LV peak A-wave KE^#^	2.5 ± 1.4^d,e^	2.3 ± 2.4^d,e^	1.9 ± 2.3	1.3 ± 0.8^a,b^	1.2 ± 1.1^a,b^	0.02

Data is presented as median ± interquartile range.

*cm/s; ^†^μJ/ml, ^**#**^mJ, LV = left ventricle, KE = kinetic energy. Superscript letters denote the different age groups, where superscript letters are used within the main body of the table, they respresent which inter-age group comparisons were statistically significant (P < 0.05).

#### Associations between 4D diastolic LV KE parameters and 2D mitral inflow diastolic parameters

**Figure 2:**
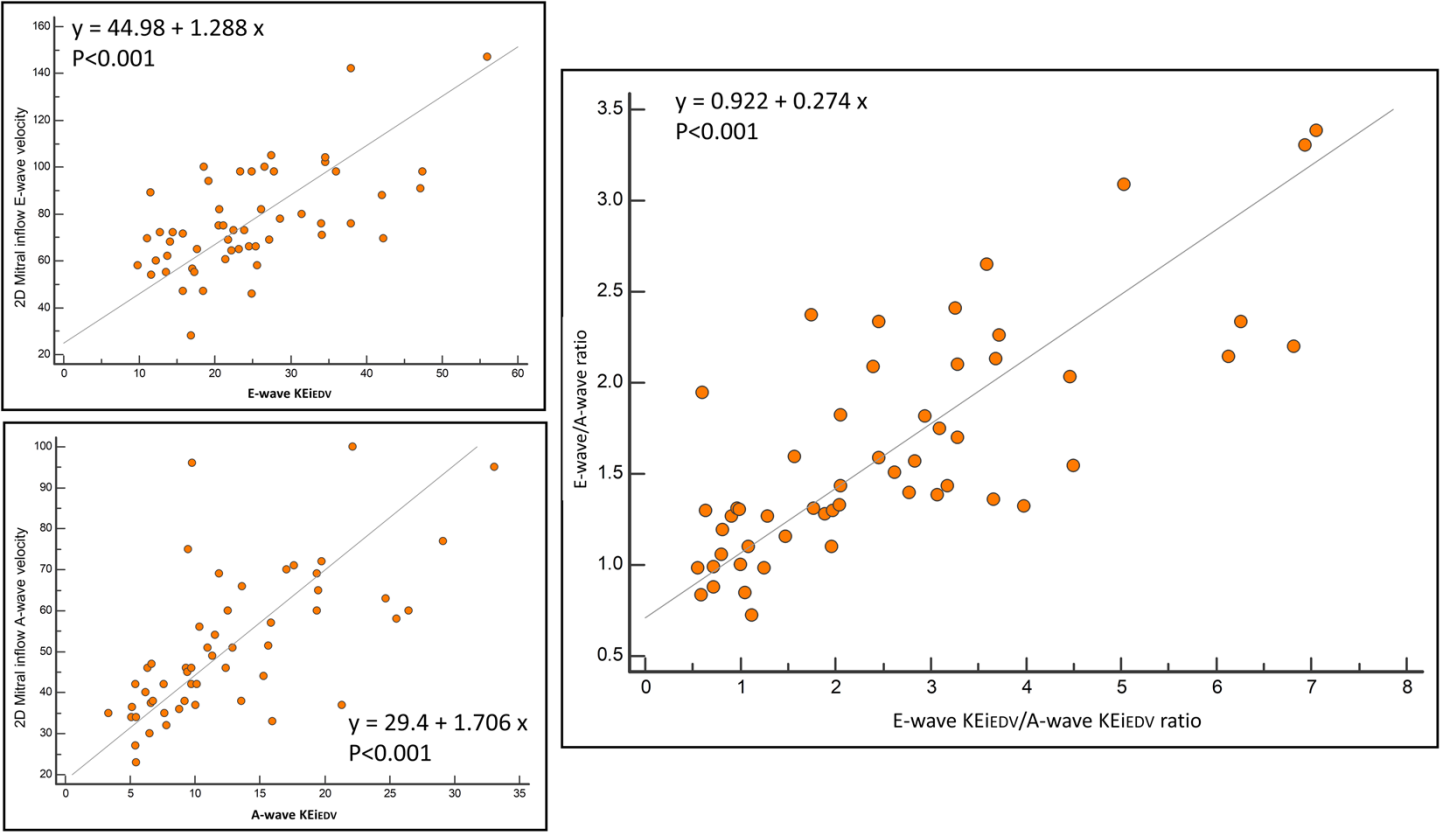
Scatter plot demonstrating the association between 2D mitral valve diastolic inflow assessments versus 4D diastolic blood flow KE parameters. Left upper box shows peak E-wave velocity vs peak E-wave KEi_EDV_, Left lower box shows peak A-wave velocity vs peak A-wave KEi_EDV_. Right box shows E/A ratio vs KEi_EDV_ E/A ratio.

The 2D measurement of E/A ratio was significantly positively correlated with the 4D diastolic measure of KEi_EDV_ E/A ratio (r = 0.77, P < 0.01) (Fig. 2). Furthermore, existing mitral inflow parameters peak E-wave and A-wave velocity were associated with their KE equivalents, peak E-wave and A-wave KEi_EDV_ (r = 0.61 and 0.66, P < 0.01 respectively).

#### Age associations

**Figure 3:**
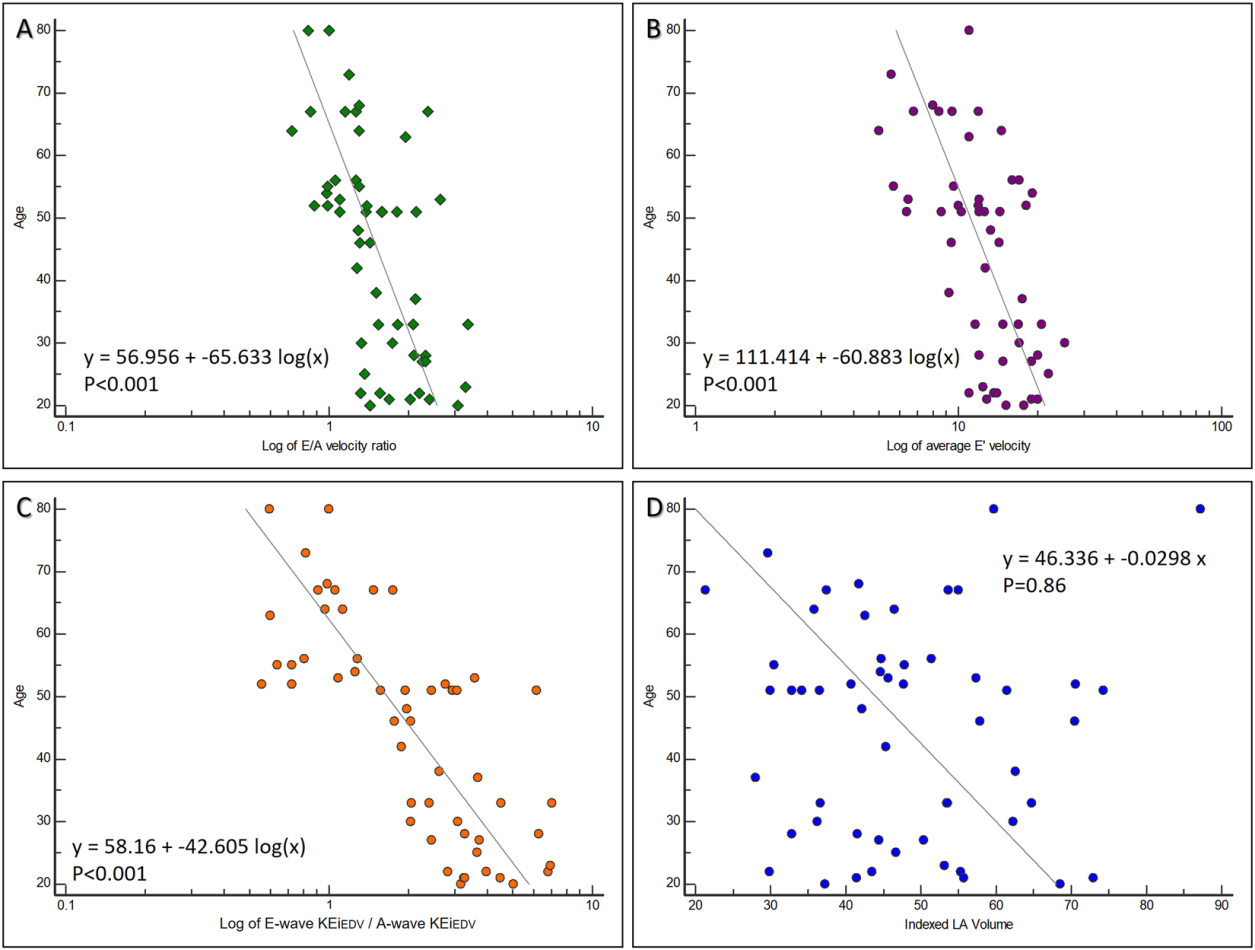
Scatter plots demonstrating the correlation between age and (**A**) log of E/A velocity ratio, (**B**) log of average E’ velocity, (**C**) log of KEi_EDV_ E/A ratio and (**D**) indexed LA volume.

Diastolic parameters E’ and E/A ratio were significantly negatively associated with age (r = −0.58, −0.627, P < 0.01), whereas E/e’ and peak A-wave velocity were positively associated (r = 0.32, 0.46, P = 0.02,<0.01 respectively) (Fig. 3, Table 3). Both A’ and peak E-wave velocity did not demonstrate significant age association (r = 0.21, −0.17, P > 0.05). Indexed left atrial volume was not associated with age (r = −0.08, P = 0.57).

**Figure 4:**
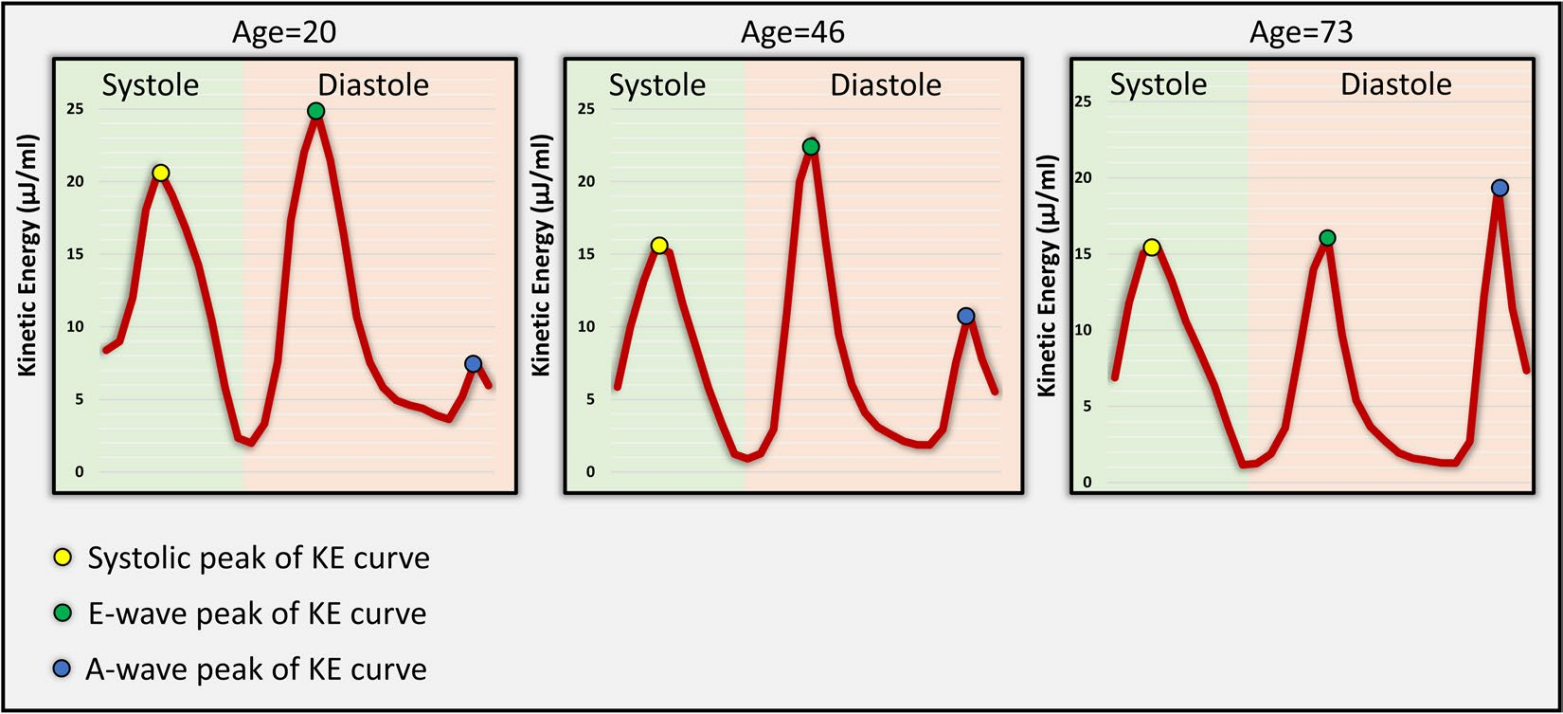
Line graph showing the various blood flow KE peaks of the cardiac cycle through systole and diastole in healthy volunteers aged 20, 46 and 73 years old. As age increases, systolic peaks decrease. In terms of diastole, early mitral inflow blood flow KE falls whilst the A-wave KEi_EDV_ sharply increases as a compensatory mechanism to maintain diastolic KE and adequate filling through physiological aging. Time across the cardiac cycle is given on the x-axis, whereas KE is given on the y-axis, in μJ/ml.

Peak E-wave KEi_EDV_ (Fig. 4), A-wave KEi_EDV_ and KEi_EDV_ E/A ratio are significantly associated with age (r = −0.51, 0.65 and −0.79, P < 0.01, respectively). All other KE parameters did not display significant age association (Table 3).

**Table 3:** Table of associations between age and standard diastolic parameters, as well as 4D diastolic LV blood flow KE parameters. The lower section of the table shows the associations between 2D and 4D parameters.

Spearman’s rho rank correlation coefficient			P-value
**Age Associated associations**			
Standard Diastolic Parameters	E’	−0.58	<0.01
	A’	0.21	0.14
	E/e’	0.32	0.02
	LV peak E-wave velocity	−0.17	0.21
	LV peak A-wave velocity	0.46	<0.01
	E/A ratio	−0.63	<0.01
	LAVi	−0.08	0.57
4D Diastolic LV KEi_EDV_ parameters	LV peak E-wave KEi_EDV_	−0.51	<0.01
	LV peak A-wave KEi_EDV_	0.65	<0.01
	KEi_EDV_ E/A ratio	−0.79	<0.01
	LV diastolic KEi_EDV_	−0.21	0.13
	LV global KEi_EDV_	−0.18	0.20
	LV systolic KEi_EDV_	−0.01	0.94
4D Diastolic LV raw KE parameters	LV global KE	−0.19	0.16
	LV systolic KE	−0.29	0.03
	LV diastolic KE	−0.1	0.47
	LV peak E-wave KE	0.09	0.51
	LV peak A-wave KE	−0.4	<0.01
**Association of 4D diastolic LV KE parameters to 2D mitral inflow parameters**			
E-wave velocity to E-wave KEi_EDV_	0.61		<0.01
A-wave velocity to A-wave KEi_EDV_	0.66		<0.01
E/A ratio to KEi_EDV_ E/A ratio	0.77		<0.01

2D = two dimensional, 4D = four dimensional, KE = kinetic energy of blood, LV = left ventricle, LAVi = left atrial volume indexed, KEi_EDV_ = kinetic energy indexed to end-diastolic volume.

#### Regression

In univariate analyses of association with age, peak E and A-wave velocities, E/A ratio, E/e’, peak E and A-wave KEi_EDV_ and KEi_EDV_ E/A ratio were all statistically significant (P < 0.05). E/A ratio and KEi_EDV_ E/A ratio had the strongest association (beta −15.39 and −6.81, P < 0.01 respectively). Table 4 provides the full results for the univariate analysis.

**Table 4:** Results for univariate and multivariate linear regression of velocity and KE parameters.

Variables	Univariate		Multivariate (stepwise)	
	Coefficient β (SE)	P-value	Coefficient β (SD)	P-value
LV peak E-wave velocity	−0.22 (0.11)	0.046		
LV peak A-wave velocity	0.40 (0.13)	0.0029		
E/A ratio	−15.39 (3.24)	<0.01		
E/e’	1.90 (0.74)	0.01	1.87 (0.5)	<0.01
LV peak E-wave KEi_EDV_	−0.90 (0.20)	<0.01		
LV peak A-wave KEi_EDV_	1.47 (0.29)	<0.01		
KEi_EDV_ E/A ratio	−6.81 (0.99)	<0.01	−6.78 (0.89)	<0.01

SE = standard error, KE = kinetic energy.

A model was created using multiple linear stepwise regression with forward elimination methods for the dependent variable, age. Peak E-wave velocity, peak A-wave velocity, E/A ratio, peak E-wave KEi_EDV_ and peak A-wave KEi_EDV_ were all excluded as non-significant (P > 0.1). The resultant model included E/e’ as well as KEi_EDV_ E/A ratio (P < 0.01), with an adjusted R^2^ value of 0.57 (residual SD = 11.3) (Table 4).

#### Intra-observer and inter-observer reproducibility

All global KE parameters demonstrated excellent concordance correlation coefficient (average 0.97, with 99% accuracy and 97% precision). The average coefficient of variability for all variables was 6 ± 2%. The coefficients of variability for intra-observer tests for LV global KE, LV systolic KE, LV diastolic KE, peak E and A wave KE were 3.5%, 3.9%, 6.0%, 4.4% and 5.5% respectively. For the same variables, the inter-observer coefficients of variability were 7%, 11%, 6.4%, 6.6% and 6.3%. The full results for intra-observer and inter-observer reproducibility are detailed in the online Supplementary File, Table 1.

## Discussion

This study has quantified 4D-flow derived diastolic KE parameters, looking specifically at their association with age as well as correlating them with traditional 2D mitral inflow measures. We have provided preliminary reference values for specific KE parameters in diastole for healthy individuals in different age groups. It appears that there are no significant haemodynamic differences between the sexes. However, these data demonstrate that specific diastolic KE parameters (namely peak E-wave and A-wave KEi_EDV_, along with KEi_EDV_ E/A ratio) not only show high correlations with existing 2D mitral inflow velocity assessments but show a stronger association to increasing age. There is a clear reduction of peak E-wave KEi_EDV_ with advancing age, coupled with a compensatory increase in peak A-wave KEi_EDV_ resulting in a progressive decline in KEi_EDV_ E/A ratio. This may provide a deeper insight into the physiological adaptations of aging in healthy individuals. In addition, the reproducibility of semi-automated KE parameters was excellent.

### Haemodynamic changes with age

The values provided for blood flow KE for various elements of diastole are consistent with existing literature^16,17^. Adding to previous data, the present study has demonstrated that as healthy individuals age, their gross diastolic KE remains stable (P > 0.05). In addition, healthy adults above the age of 50-years had a significantly lower KEi_EDV_ E/A ratio than all other younger age groups. This can be explained by a compensatory increase in peak A-wave KEi_EDV_, as peak E-wave KEi_EDV_ steadily declines. Progressive reduction in peak E-wave KEi_EDV_ with age is explained by impaired myocardial relaxation which increases myocardial stiffness. Our results support existing knowledge that the contribution of the atrial systole to the LV blood pool volume is less in younger individuals compared with older individuals^18^.

Research by Wong *et al*. supports the finding that peak E-wave LV blood flow KE declines with age^19^, despite differences in study design to the present study. The current study is the first to compare 4D blood flow LV energetics with existing 2D standard mitral inflow metrics of diastolic function for their association to advancing age. Wong *et al*.'s study included paediatric healthy individuals, whilst the present study was focused specifically on an adult population only (20–80 years). In their study 4D flow CMR acquisition used prospective ECG-gating with *kt* acceleration, which results in temporal blurring during late diastole^20^. Even with these differences, peak E-wave KEi_EDV_ shows a consistent negative correlation with age in both studies (r^2^ = 0.545, P < 0.0001 vs r = −0.51, P = 0.0001). Furthermore, earlier 4D flow work has demonstrated that older individuals display fewer LV diastolic vortices than younger patients, in addition to a reduction in vortex velocity^21^. This is consistent with the present study, as older individuals have a lower peak E-wave KEi_EDV_, resulting in additional work from the atrium to restore this imbalance. These changes supplement existing literature that suggests that there are numerous cardiac changes that occur with physiological aging^22^.

### Association of 2D and 4D parameters with age

With increasing age, the stages of diastole alter in an adaptive manner to maintain LV filling. This is the first study to directly compare both existing retrospectively tracked, 2D mitral inflow parameters with 4D blood flow KE parameters in diastole. Although LV haemodynamics can be quantified using velocity measurements; the present study suggests that a 4D flow CMR-derived assessment provides a closer association with the changes seen with age. This finding is striking, and true for the blood flow KE of early and late mitral filling as well as KEi_EDV_ E/A ratio versus similar 2D through-plane mitral inflow metrics. This may be explained by the fact that 4D flow CMR derived LV blood flow KE metrics are more closely associated with myocardial relaxation coupled with its resulting haemodynamic forces.

### 2D inflow metrics versus 4D LV blood flow energetics

In this study, there is a strong correlation between 2D mitral inflow metrics and 4D blood flow energetics. This is because during both early and late filling phases of diastole, a large proportion of blood flow kinetic energy occurs in the mitral through-plane. However, 4D flow KE metrics demonstrate a stronger association with adaptive changes seen in age, plausibly because of two reasons. Firstly, 4D blood flow energy assessment includes not only mitral inflow but also the KE energy in the LV vortex. Previous studies have demonstrated that the diastolic vortex is responsible for a significant fraction of LV filling volume^23^. Thus, intraventricular fluid mechanics are an important determinant of global chamber LV operative stiffness. Hence, plausibly vortex KE is associated with LV relaxation. Secondly, this study demonstrates very high accuracy and precision of 4D LV blood flow energetics which will reduce bias when investigating age related association. Hence, it is reasonable to conclude that the 4D LV blood flow energetics offer enhanced assessment of flow changes associated with impaired LV relaxation. Multivariate linear regression demonstrates that both E/e’, a marker of myocardial relaxation, and KEi_EDV_ E/A ratio are the most independently associated variables with aging. 4D flow CMR techniques such as retrospective valve tracking have been shown to be both highly accurate and reliable^24,25^ superseding through plane motion issues seen with 2D valvular quantification techniques. A KE evaluation may prove to be the more effective given the fact that it incorporates all of the 3D LV blood flow data.

### Clinical perspective

Assessment of cardiac haemodynamics plays an important role in routine assessment of patients presenting with shortness of breath and possible heart failure. Two-dimensional mitral inflow metrics are heavily dependent on pre-loading conditions making them unreliable for the assessment of LV diastolic function. Novel semi-automated technologies, such as 4D flow CMR derived blood flow KE of the LV, can offer a highly accurate and precise plus more comprehensive evaluation of cardiac haemodynamics.

From this study’s results, it is evident that a semi-automated analysis of KE is not only reliable to quantify diastolic function similar to previous 2D mitral inflow methods, but in addition, it appears that 4D flow-derived KE parameters show increased associations with age. Not only does this allow a more accurate measurement of the normal age-associated adaptation of left ventricular diastolic function, but this non-invasive technique may enable a more precise categorization of impaired filling within disease states.

This study does not informs us about the influence of pre-loading condition on LV blood flow KE. We speculate that LV blood flow diastolic KE indices may be less susceptible to pre-load than peak velocity inflow velocity assessment as they factor in velocity profile of the whole blood flow in the LV including the vortex and other ancillary flow during diastole.

### Study limitations

 Respiratory navigation was omitted for the 4D flow acquisition which could have influenced KE parameters. However, whole-heart 4D flow head-to-head comparison studies have also demonstrated that non-respiratory navigated acquisition of 4D flow is comparable to respiratory navigated acquisition for intra-cardiac KE quantification^26^. In addition, a recent study validated a non-respiratory navigated 4D Flow EPI acceleration sequence for clinical use^20^. The temporal resolution of the 4D flow was 40 ms, which may affect the quality of KE assessment. The LV geometry was defined from a stack of LV cines acquired during breath-holding while the 4D flow was acquired during free breathing. Hence, although spatial mis-registration was corrected for, other issues still remain including difference in heart rate and physiological conditions. This may have impacted on the time-varying flow characteristics which could not be corrected for. Results from this study cannot be applied to patients with significant valvulopathy, cardiomyopathies or congenital heart disease.

## Conclusions

Increasing age results in a steady decline in peak E-wave KEi_EDV_ accompanied by an increase in peak A-wave KEi_EDV_. These elements of diastole were highly associated with age, demonstrating significance across all age groups. Moreover, KE parameters consistently showed a stronger association to age than existing methods of diastolic evaluation, suggesting that their use may be able to more accurately track declines in left ventricular diastolic function. In addition, semi-automated, LV blood flow KE mapping demonstrated a high degree of reproducibility, facilitating future transitions to clinical practice. Further studies utilizing patient populations are necessary to validate these preliminary findings and investigate if LV energetics are less susceptible to LV loading conditions.

## Methods

### Study Population

Healthy adult volunteers between the ages of 20 to 80 years old, were prospectively recruited from two centers: Leeds, UK and Leiden, Netherlands. They had no history or symptoms of cardiovascular disease, were not on cardiovascular or other relevant medication and had no contraindications to CMR.

The study population was divided into five comparably-sized adult agegroups. 

The study protocol was approved by the National Research Ethics Service (12/YH/0169) in the UK and the institutional Medical Ethical Committee (P11.136) in Leiden. The study complied with the Declaration of Helsinki and all patients gave written informed consent.

### CMR protocol and Image acquisition

CMR was performed on a dedicated cardiovascular 1.5 Tesla Philips Ingenia system equipped with a 28-channel coil and Philips dStream digital broadband MR architecture technology.

The CMR protocol included the following:

1. Survey images
2. Cine imaging: vertical long-axis, horizontal long-axis, 3-chamber (LVOT-views), and LV volume contiguous short axis stack. All cines were acquired with a balanced steady-state free precession (bSSFP), single-slice breath-hold sequence. Typical parameters for bSSFP cine were as follows: SENSE factor 2, flip angle 60°, echo time (TE) 1.5 milliseconds, repetition time (TR) 3 milliseconds, field of view 320–420 mm according to patient size, slice thickness 8 mm, and 30 phases per cardiac cycle.
3. For whole heart 4D flow, field of view (FOV) was planned in trans-axial plane making sure whole heart was in FOV. If the necessary number of slices was increased. 4D flow was done using fast field echo (FFE) pulse sequence (EPI based, 3D) with retrospective ECG-triggering. The acquisition voxel size was kept as close as possible to 3 × 3 × 3 mm^3^. Field-of-view and number of slices (i.e., the 3D volume) was adapted to the subject’s size. The standard scan parameters were: echo time 3.5 ms, repetition time 10 ms, flip-angle 10°, field-of-view 400 mm, number of signal averages 1. VENC 150 cm/sec. Acceleration was achieved by Echo Planar Imaging with EPI factor 5. Free breathing was allowed, and no respiratory motion compensation was used. Number of slices was 40 with a temporal resolution of 40 ms. The number of reconstructed phases was set to 30. The 4D flow encoding was performed by standard 4-point encoding.

### 4D flow error corrections and quality checks

Online/offline 4D flow data quality assurance checks were done as per previously published literature^20^. The effects of concomitant gradient terms were compensated using Maxwell correction methods by the CMR scanner. Remaining background errors were corrected by the local phase correction (LPC) filter on the CMR scanner performed in two-dimensional way - slice by slice. The LPC is a magnitude-weighted spatial low pass filter; pixels that are expected to be part of the static background are used with a higher weight than noisy background pixels or pixels that are expected to contain flow to determine the local phase offset. LPC uses surrounding tissue to determine “static” areas^27,28^.

All three-directional phase contrast data sets were investigated for phase aliasing artefacts. If present then phase unwrapping was performed as per previously published guidelines on phase-contrast methods^29^. Additionally, any spatial misalignment of 4D flow data to cine imaging was corrected before any flow analysis was performed. This was done by visualizing streamlines in 4-chamber view at peak systole and repositioning them over descending aorta. Similar checks were done during diastole in 4-chamber and 2-chamber views for peak mitral inflow streamlines.

### Image analysis

All images were analysed by PG (3 years experience in advanced CMR techniques, SC (1-yearexperience in advanced CMR techniques) and RVDG (>5 years experience in advanced CMR techniques, RVDG did the blinded LV flow KE mapping). Images were evaluated offline using research software (MASS; Version 2016EXP, Leiden University Medical Center, Leiden, The Netherlands). Left ventricular volumes and EF were determined according to standard methods. Peak mitral early diastolic annular velocity (e’) measurements were recorded as per previously published methods^30^. Left atrial volume was measured in 2-chamber and 4-chamber cines using published techniques^31^.

### 2D mitral inflow metrics

Phase unwrapping was performed on source images when aliasing occurred in the area of interest as per previously published guidelines on phase-contrast methods^29^. All 2D mitral inflow flow assessments were done using validated techniques including retrospective valve tracking, with measurement planes positioned perpendicular to the inflow direction on two- and four-chamber cines^25,32,33^. Background velocity correction (i.e., for correction of through-plane motion and phase offset) was used from the velocity sampled in the myocardium in the reformatted dynamic phase contrast plane. Contour segmentation was performed manually. Mitral inflow metrics computed included: peak early mitral inflow velocity (E-wave velocity), peak late mitral inflow velocity (A-wave velocity) and E/A ratio.

### 4D LV kinetic energy mapping

For calculation of LV blood flow KE parameters, the LV volumetric mesh was resliced into short-axis sections of 2 mm thickness and pixel spacing equal to the original reconstructed pixel size of the short-axis cine acquisition (1.0–1.2 mm). This time-resolved, high-resolution LV mesh is constructed by representing the mesh in cylinder coordinates. The LV radius for a given angle and LV level is derived by linear interpolation. This time-resolved LV mesh was applied on the raw velocity-encoded data as previously described^34^. Correction for translational and rotational misalignment between the short-axis cine and the 4D Flow CMR acquisition was performed using automated image registration as previously described^35^. This was done using automated image registration using Elastix^36^. It was performed between cine short‐axis data and velocity magnitude reconstructed images of the 4D flow data using a single phase that visually showed the best depiction of the LV in the velocity magnitude 4D flow image. Registration was restricted to translation only. This registration result was then propagated to all 4D flow phases. Registered 4D flow MRI contours were then visually reviewed for any possible registration or projection errors and manually corrected whenever needed.

For each volumetric element (voxel) the KE was computed using the following formula:

$${\bf{K}}{\bf{E}}=\frac{{\bf{1}}}{{\bf{2}}}{{\boldsymbol{\rho }}}_{{\boldsymbol{blood}}}\cdot {{\boldsymbol{V}}}_{{\boldsymbol{voxel}}}\cdot {{{\boldsymbol{v}}}_{{\boldsymbol{voxel}}}}^{{\bf{2}}}$$

with ${\rho }_{blood}$ being the density of blood (1.06 g/cm^3^), ${V}_{voxel}$ the voxel volume and ${v}_{{\boldsymbol{voxel}}}$ the velocity magnitude of the corresponding voxel. For each phase, the total KE within the LV was obtained by summation of the KE of every voxel. All KE parameters were normalized to the LV end-diastolic volume (KEi_EDV_) and accordingly reported in μJ/ml. Time-resolved kinetic energy curves were generated to derive physiologically relevant parameters, including: global LV KEi_EDV_ (the mean KE of LV blood flow throughout the entire cardiac cycle), systolic KEi_EDV_ (the KE of the LV blood flow during systole), diastolic KEi_EDV_ (the KE of the LV blood flow during diastole), peak E-wave KEi_EDV_ (the peak KE of the LV blood flow during early mitral filling), peak A-wave KEi_EDV_ (The peak KE of the LV blood flow during late mitral filling) and KEi_EDV_ E/A ratio (the ratio of LV peak E-wave KE to LV peak A-wave KE).

### Intra-/inter-observer reproducibility

For inter-observer reproducibility, SC and RVDG contoured the short-axis LV cine volumetric stack in 20 random study subjects and were blinded to each other’s analysis. Automated KE parameters were again generated using the new endocardial contours. For intra-observer reproducibility, SC re-analysed the LV short-axis cines for the same 10 subjects after 3 months. Akin to inter-observer reproducibility, automated KE parameters were generated using the new endocardial contours by the same observer.

### Statistical analysis

Statistical analysis was performed using IBM SPSS® Statistics 21.0. Continuous measurements are presented as mean ± standard deviation. Normality of data was tested by the Shapiro–Wilk test. Quantitative flow imaging parameters were expected to be non-parametric and were presented as median and inter-quartile ranges (IQR). Demographic comparisons were performed with an independent samples t-test. Intra-/inter-observer reliability tests were done by coefficient of variability. In different quartiles of age group, post hoc analysis was done by Kruskal-Wallis H test. Association of age to KE parameters was done by Spearman’s rank correlation coefficient test. In multi-variate analysis, a forward-conditional method was used for regression and parameters with statistical significance from one-way analysis (p < 0.05) were chosen for multi-variate analysis. A p-value < 0.05 was considered statistically significant.

### Power Calculations

Informed from previous KE studies, for the inter-age group comparisons, we expected to see a mean difference of 6.2 mJ in peak early mitral inflow KE and standard deviations of 4.2 and 4.6. On these assumptions, we need to recruit at least 9 volunteers in each group. For reproducibility tests, with an expected correlation coefficient of 0.97, we will need to do minimum of 5 cases to demonstrate reproducibility. However, to increase the clinical significance, we aim to do 10 cases for intra-observer reproducibility and 20 cases for inter-observer reproducibility.

## Electronic supplementary material

Supplementary Dataset 1
