# Supplementary material for: Impact of Age and Diastolic Function on Novel, 4D flow CMR Biomarkers of Left Ventricular Blood Flow Kinetic Energy
Source: Sci Rep. 2018 Sep 26;8:14436. doi: 10.1038/s41598-018-32707-5 (PMC6158175; doi:10.1038/s41598-018-32707-5)
Supplement: Supplementary file 1 — Supplementary Dataset 1 [file 41598_2018_32707_MOESM1_ESM.pdf]

Impact of Age and Diastolic Function on Novel, 4D flow CMR Biomarkers of Left Ventricular Blood Flow Kinetic Energy.

Saul Crandon[1], Jos J M Westenberg[3], Peter P Swoboda[1], Graham J Fent[1], James R J Foley[1], Pei G Chew[1], Louise A E Brown[1], Christopher Saunderson[1], Abdallah Al-Mohammad[4], John P Greenwood[1], Rob J van der Geest[3], Erica Dall'Armellina[1], Sven Plein[1], *Pankaj Garg[1,4].

[1]Leeds Institute of Cardiovascular and Metabolic Medicine, University of Leeds, Leeds, UK

[2]Department of Radiology, Leiden University Medical Center, Leiden, The Netherlands

[3]Sheffield Teaching Hospitals NHS Foundation Trust, Sheffield, UK

[4]Department of Infection, Immunity & Cardiovascular Disease, University of Sheffield, Sheffield, United Kingdom

Supplementary File

S Table 1. Intra-observer and inter-observer global KE parameters.

**Subject**	**BIAS (%)**	**LL (%)**	**UL (%)**	**CoV (%)**	**95% CI**	**CCC**	**ρ**	**C_b**	***P-value**
Intra-observer (n=10)									
LV KE	0.5	-9.7	10.8	3.5	0 - 5.5	0.99	99%	99%	P<0.0001
LV systolic KE	2.8	-7.2	12.8	3.9	2.6-4.9	0.99	99%	99%	P<0.0001
LV diastolic KE	-0.8	-18.7	17.2	6.0	0 - 9	0.98	99%	99%	P<0.0001
E-wave KE	0.8	-13.6	15.2	4.4	0 - 6.6	0.99	99%	99%	P<0.0001
A-wave KE	1.7	-17.1	20.5	5.5	0 - 8.4	0.99	99%	99%	P<0.0001
Inter-observer (n=20)									
LV KE	-3.4	-22	15.8	7	0 - 11	0.95	95%	99%	P<0.0001
LV systolic KE	-4.7	-35	26	11	0 - 16	0.89	90%	99%	P<0.0001
LV diastolic KE	-2.9	-20	14	6.4	1.5 - 9	0.96	97%	99%	P<0.0001
E-wave KE	-2.2	-20.6	16.3	6.6	0 - 10	0.96	96%	99%	P<0.0001
A-wave KE	-4.3	-20	12	6.3	1.9 - 8	0.98	98%	99%	P<0.0001

*for correlation
C_b=accuracy, CoV= coefficient of variability in percentage, CI=confidence interval, CCC= concordance correlation coefficient, LV=left ventricle, LL=lower-limit of agreement, UL=upper limit of agreement, ρ=precision.
